# Conditional loss of IKKα in Osterix + cells has no effect on bone but leads to age-related loss of peripheral fat

**DOI:** 10.1038/s41598-022-08914-6

**Published:** 2022-03-22

**Authors:** Jennifer L. Davis, Nitin Kumar Pokhrel, Linda Cox, Nidhi Rohatgi, Roberta Faccio, Deborah J. Veis

**Affiliations:** 1 Musculoskeletal Research Center, Division of Bone and Mineral Diseases, Washington University School of Medicine, St. Louis, MO 63110 USA; 2 Department of Pathology and Immunology, Washington University School of Medicine, St. Louis, MO 63110 USA; 3 Musculoskeletal Research Center, Department of Orthopedic Surgery, Washington University School of Medicine, St. Louis, MO 63110 USA; 4 Shriners Hospitals for Children, St. Louis, MO 63110 USA

**Keywords:** Physiology, Endocrinology, Pathogenesis

## Abstract

NF-κB has been reported to both promote and inhibit bone formation. To explore its role in osteolineage cells, we conditionally deleted IKKα, an upstream kinase required for non-canonical NF-κB activation, using *Osterix (Osx)-Cre*. Surprisingly, we found no effect on either cancellous or cortical bone, even following mechanical loading. However, we noted that *IKKα* conditional knockout (cKO) mice began to lose body weight after 6 months of age with severe reductions in fat mass and lower adipocyte size in geriatric animals. qPCR analysis of adipogenic markers in fat pads of cKO mice indicated no difference in early differentiation, but instead markedly lower leptin with age. We challenged young mice with a high fat diet finding that cKO mice gained less weight and showed improved glucose metabolism. Low levels of recombination at the* IKKα *locus were detected in fat pads isolated from old cKO mice. To determine whether recombination occurs in adipocytes, we examined fat pads in *Osx-Cre;TdT* reporter mice; these showed increasing *Osx-Cre*-mediated expression in peripheral adipocytes from 6 weeks to 18 months. Since *Osx-Cre* drives recombination in peripheral adipocytes with age, we conclude that fat loss in cKO mice is most likely caused by progressive deficits of IKKα in adipocytes.

## Introduction

Although NF-κB is primarily considered key to acute inflammatory responses, this is not universally true, particularly for the alternative or non-canonical pathway. Unlike the canonical pathway, which is activated in minutes and generally inactivated within hours, alternative NF-κB is induced over many hours and typically persists for days. NIK functions as a central signaling component in this pathway, orchestrating signals from multiple stimuli and activating the downstream kinase IKKα. This triggers phosphorylation of p100 and its partial processing, subsequently leading to persistent activation of the p52/RelB transcriptional complex^1^. Besides participating in inflammatory responses due to its activation in immune cells, alternative NF-κB is involved developmentally in lymph node organogenesis, via the stroma^2–4^, and plays a cell-extrinsic role in myelopoiesis^5^. Expression of NIK in intestinal epithelial cells controls specialized antigen-presenting cells in the gut^6^. Outside of its effects on the immune system, alternative NF-κB has been shown to control pathologic angiogenesis via direct actions in endothelial cells^7^. Upregulation of NIK in skeletal muscle occurs in patients with metabolic syndrome and decreases with weight loss after gastric bypass^8^. Alternative NF-κB also plays a role in metabolism via direct actions in pancreatic beta cells and hepatocytes^9^. Thus, there is ample evidence that the alternative NF-κB pathway is important in a variety of cell types and physiologic contexts.

Bone is a dynamic organ, maintained by the coordinated actions of osteoblasts, which produce bone matrix, osteoclasts, which degrade bone, and osteocytes, which act as mechanosensors directing osteoblast and osteoclast activities. Osteocytes differentiate from osteoblasts, and together these cell types comprise the osteolineage. Few studies, all employing global knockout models, have directly addressed the role of alternative NF-κB in osteoblasts^10–12^. These displayed complex skeletal phenotypes, largely pointing to positive effects on bone mass with pathway inhibition. However, we recently explored the role of alternative NF-κB in bone using a constitutively active NIK allele (NT3) lacking a negative regulatory domain, expressed in the osteolineage, finding increased bone mass^13^. In contrast, the effect of alternative NF-κB signaling in the osteoclast is well established. We and others have previously shown that NIK, IKKα, and RelB support osteoclastogenesis, particularly during pathological osteolysis^12,14–20^. Due to these direct effects of alternative NF-κB on osteoclasts and physiologic coupling between osteoclasts and osteoblasts, the cell autonomous role of this pathway in osteoblasts remains unclear.

To better understand the role of alternative NF-κB in bone formation, we set out to conditionally inhibit it in osteoblasts. As in the previous study with activated NIK, we chose to target early osteoblasts for modulation of alternative NF-κB throughout the lifespan of osteoblasts and osteocytes. The transcription factor *Osterix*, also known as *Sp7*, is upregulated as mesenchymal stromal cells become committed to the osteoblast lineage, and in adult mice, its expression in the skeleton is largely confined to osteoblasts and most osteocytes^21,22^. Therefore, the *Osterix* promoter has been widely used to drive *Cre* expression in many studies of bone^23,24^. At the initiation of this study, mice with a conditional allele for deletion of NIK were not yet available, so we employed *IKKα*^*fl/fl*^ mice, ablating the second kinase in the alternative NF-κB pathway.

## Results

### Conditional deletion of *IKKα* in the osteoblast lineage does not alter bone mass

We mated *IKKα*^*fl/fl*^ and *Osx-Cre* mice to generate *Osx-Cre;IKKα*^*fl/fl*^ (cKO) and *Cre-negative* (CON) littermates. Dams were kept on doxycycline throughout pregnancy and until pups were weaned to prevent early Cre expression that can impact skeletal growth. Activation of Cre and excision of the floxed *IKKα* allele were assessed in bone marrow-derived mesenchymal stromal cell cultures (BMSCs) under osteogenic conditions and in flushed, crushed bones from cKO and CON littermates. *Cre* expression was undetectable in unstimulated BMSCs and rose during osteogenesis (Fig S1a). Interestingly, however, recombination of the *IKKα* allele was robust, even prior to addition of osteogenic media (Fig S1b). Importantly, *Cre* expression and recombination did not occur in CON osteoblasts or bones but were readily detected in cKO samples (Fig S1b–d). We next assessed the effect of *IKKα* deficiency on osteogenesis in vitro and found a modest increase in mineralization as well as expression of osteoblast markers (Fig S2).

**Figure 1:**
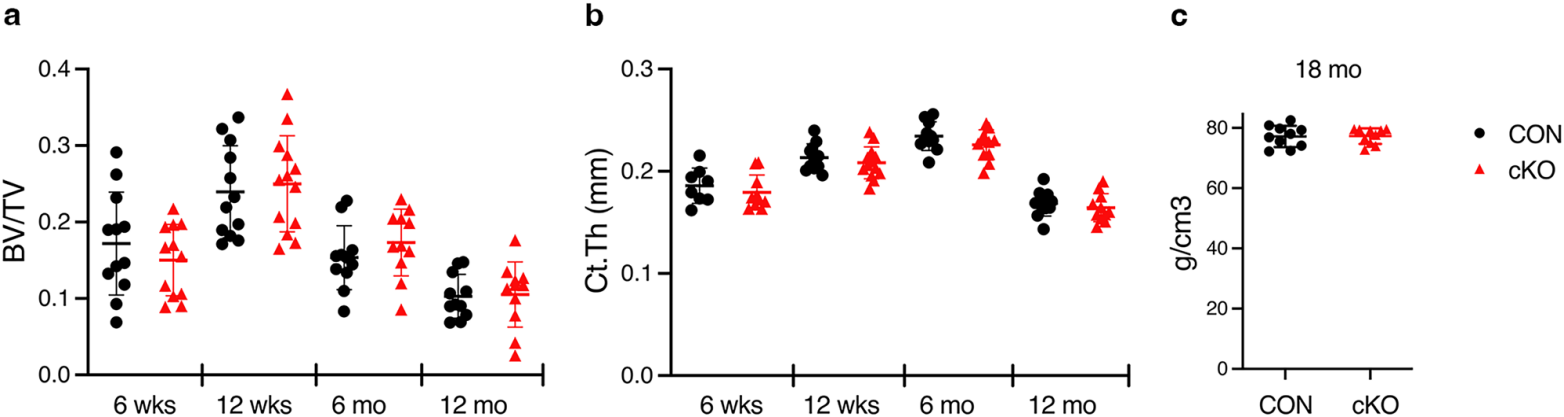
*IKKα* cKO mice have similar basal bone mass compared to controls. (**a**) Cancellous bone volume fraction (BV/TV) or (**b**) Cortical thickness (Ct.Th) of tibiae at 6 wks (n = 12), 12 wks (n = 11–13), 6 mo (n = 11), 12 mo (n = 11) by in vivo microCT. (**c**) Whole body bone mineral density by dual-energy x-ray absorptiometry (DXA) for 18–20 mo mice (n = 11). Data are represented as mean ± SD. CON = black circles, cKO = red triangles, all male mice. Non-significant between genotypes at each age by student’s, unpaired, two-tailed t-test.

**Figure 2:**
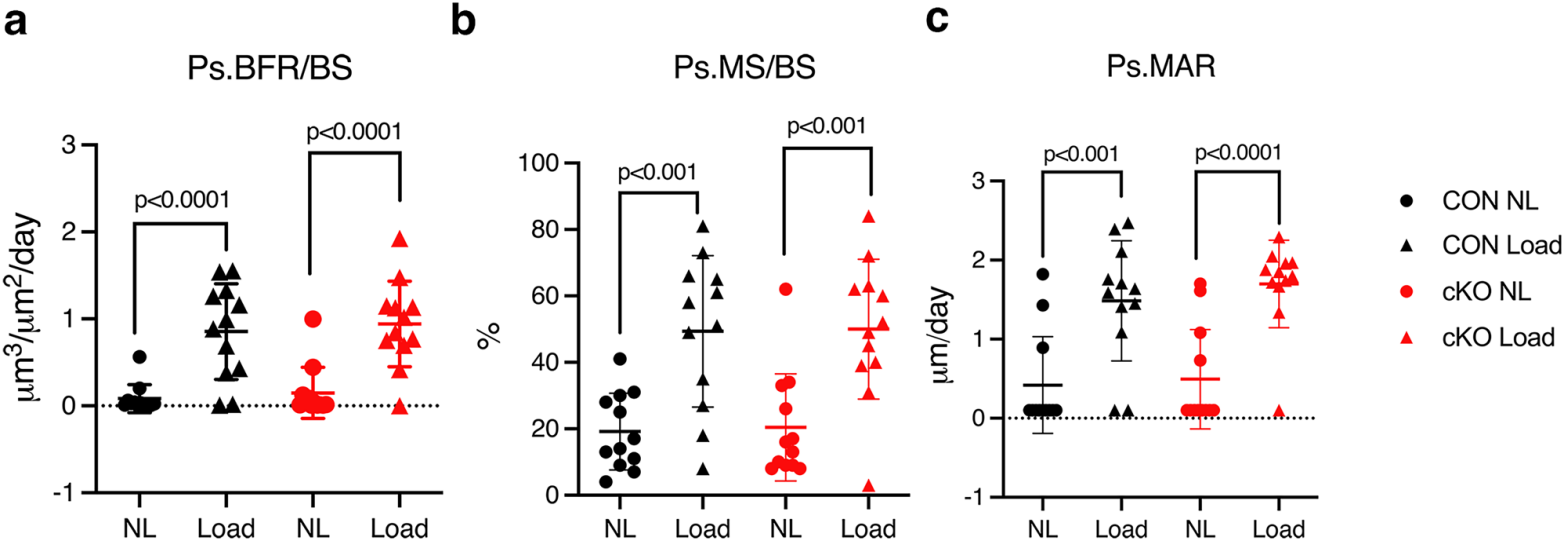
*IKKα* cKO mice have a similar response to anabolic loading compared to control. Anabolic response to unilateral axial tibial compression was assessed by dynamic histomorphometry, measuring parameters along the periosteum (Ps) after 2 wks. (**a**) Bone formation rate per bone surface (Ps.BFR/BS), (**b**) Mineralizing surface per bone surface (Ps.MS/BS), and (**c**) Mineral apposition rate (Ps.MAR). CON = black, cKO = red, all male mice. Right tibiae were loaded (Load, triangles) and left tibiae served as non-loaded (NL, circles) controls. Results are presented as mean ± SD. n = 12 per genotype. 2-way ANOVA followed by Tukey’s multiple comparison test; response to load within genotypes. There were no significant differences between genotypes, or interactions between genotype and loading.

We used in vivo microCT to screen for bone effects in male mice at multiple ages and found no differences in cortical or cancellous parameters in the tibia at any time (Fig. 1a, b, Figs S3–S6). Aged males were also screened by DXA, but again no differences in bone mass were seen (Fig. 1c). In females, ex vivo microCT at 16 weeks of age also failed to detect bone changes after *IKKα* deletion (Fig S7). To determine if an anabolic condition would elicit a bone-specific role for IKKα, we applied unilateral tibial compression for 2 weeks in skeletally mature, 16 week old male mice. However, again, we saw no differences by genotype (Fig. 2). We therefore concluded that IKKα plays little or no role in osteogenesis in basal or mechanical loading conditions.

### Aging *IKKα* cKO mice lose fat, associated with improved glucose metabolism

**Figure 3:**
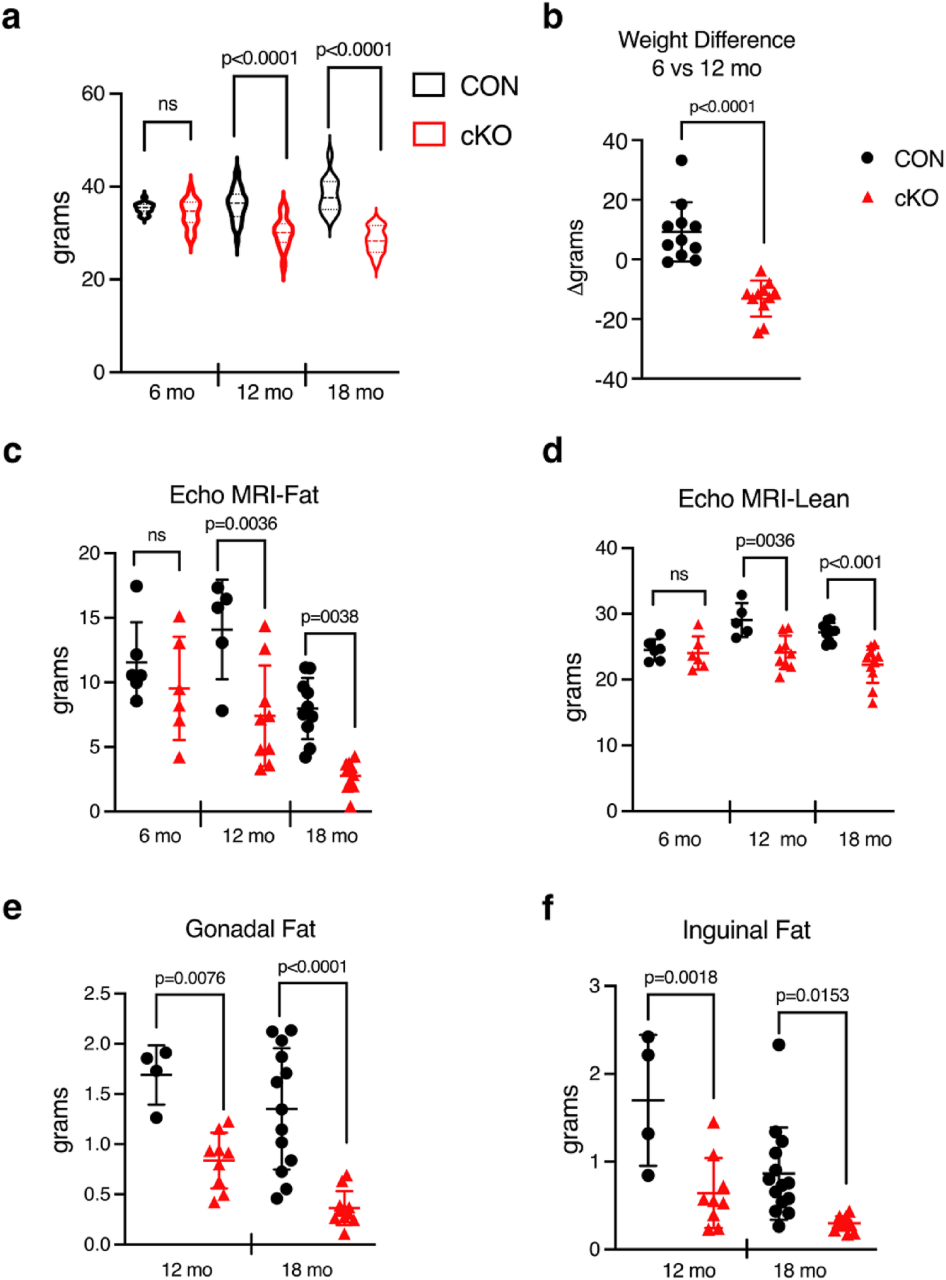
*IKKα* cKO mice have attenuated weight gain, lower total fat, and less lean mass with age. (**a**) Body weights at 6 mo (n = 15–16), 12 mo (n = 28–31), 18 mo (n = 11–13). (b) Weight change (%) from 6 to 12 mo (n = 11). EchoMRI measurement of (**c**) total fat mass and (**d**) total lean mass at 6mo (n = 6), 12mo (n = 5–9), 18mo (n = 10–11). (**e**) Gonadal and (**f**) Inguinal fat pad weights at 12 mo (n = 4–9) and 18 mo (n = 11–14). Data are represented as mean ± SD, all male mice. CON = black, cKO = red. Student’s, unpaired, two-tailed t-test (for b) or 2-way ANOVA followed by Sidak multiple comparisons test (cross genotype comparisons): ns – non-significant.

As we aged mice to examine their bone phenotype, we noted that the cKO mice appeared smaller. Indeed, analysis of body weights showed not only lower weights for the cKO cohorts overall at both 12 and 18 months, but also a 13% decrease in weight in the same animals between 6 and 12 months of age, compared to a 9% increase over the same period in CON (Fig. 3a, b). EchoMRI was then used to quantitate fat and lean mass. cKO mice displayed distinctly lower fat mass at 12 and 18 months (47% and 62% respectively), with a more modest, but still statistically significant, decrease in lean mass (Fig. 3c, d). Post mortem, both gonadal and inguinal fat pads were smaller in cKO than CON mice (Fig. 3e, f).

**Figure 4:**
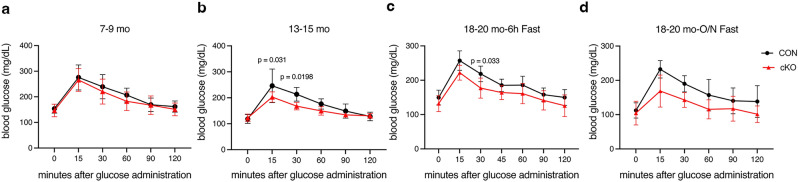
Older *IKKα* cKO mice show improved glucose tolerance. Glucose Tolerance Test (GTT) was performed after a 6 h fast at all ages and overnight (O/N) at 18–20 mo. CON = black, cKO = red, all male mice. Blood glucose measurements at (**a**) 7–9 mo (n = 7–9), (**b**) 13–15 mo (n = 5–6), (**c**) 18–20 mo (n = 4–7), and (**d**) 18–20 mo after O/N fast (n = 4–7). Data are represented as mean ± SD. Repeated measures 2-way ANOVA followed by Sidak multiple comparisons test, with p values indicated where significant.

Because reductions in fat are often accompanied by changes in glucose metabolism, we challenged mice with glucose tolerance testing (GTT) after a 6 h fast. At 7–9 mo of age, the response to glucose challenge was nearly identical (Fig. 4a). By middle-age (13–15 mo), cKO mice showed a mild but significant decrease in peak blood glucose levels (Fig. 4b). Old (18–20 mo) cKO animals displayed a trend towards lower peak glucose (Fig. 4c), and this difference was further accentuated after an overnight fast (Fig. 4d), indicating improved glucose tolerance in aged cKO mice.

### *Osx-Cre* mediates increasing recombination in adipocytes with age

**Figure 5:**
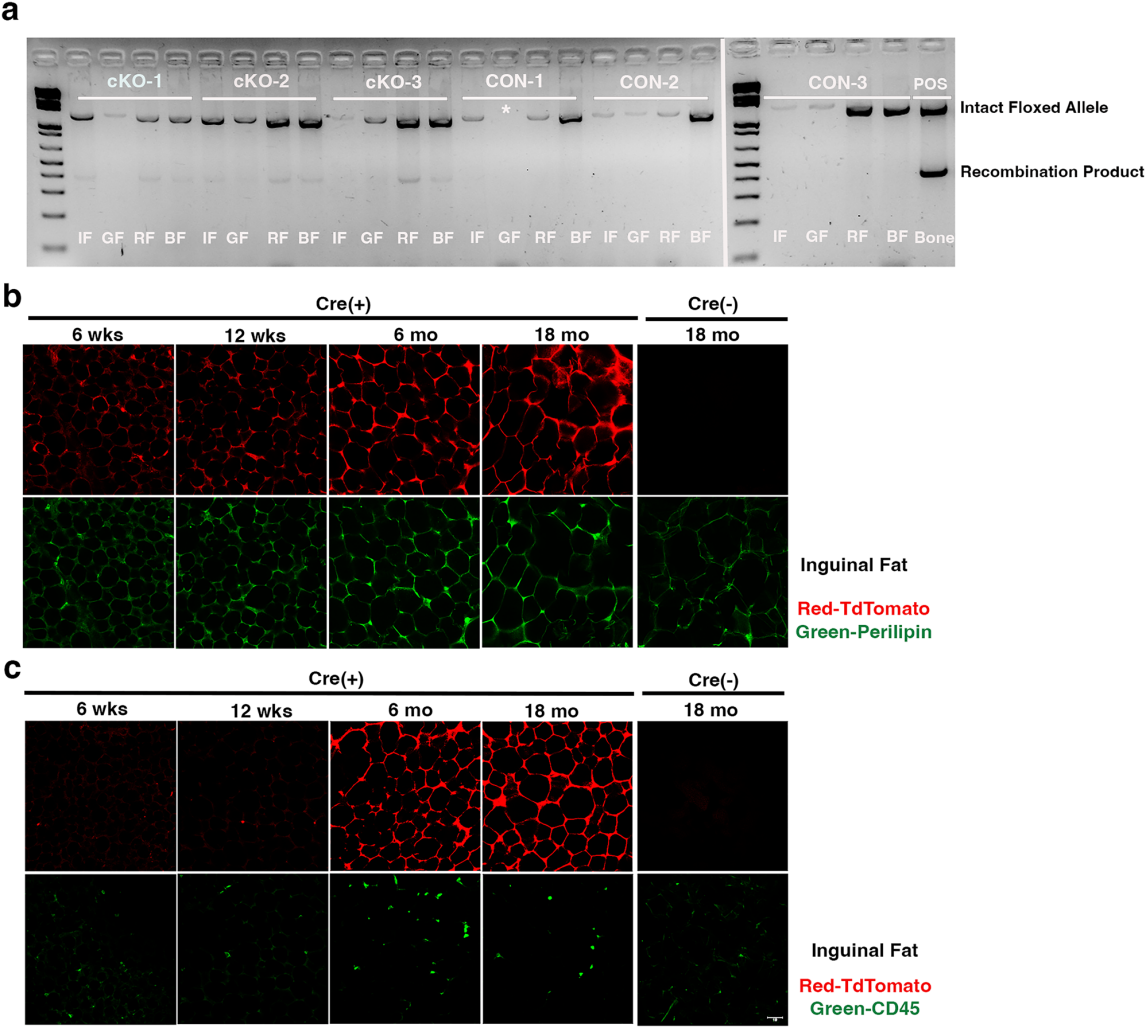
*Osx-Cre* mediates recombination in peripheral adipocytes. (**a**) PCR of genomic DNA from inguinal fat (IF), gonadal fat (GF), renal fat (RF), and brown fat (BF), isolated from 15 mo male mice. Whole, flushed bone was used as a positive control (POS). Intact floxed allele = 1.3 kb and recombination product = 460 bp. *, sample lost in loading. Right panel was originally on the bottom row of the same gel as the left panel. n = 3 biological replicates. Representative immunofluorescence staining for (**b**) Perilipin (green) or (**c**) CD45 (green) in inguinal fat from *Osx-Cre;TdT* reporter mice (red) at 6 wks, 12 wks, 6 mo, 18 mo, or *Osx-Cre* control mice at 18 mo.

Based on these changes in fat mass and glucose metabolism, we next sought to determine if the *IKKα* floxed locus was recombined in cKO peripheral fat. We performed PCR to detect the deleted allele in inguinal, gonadal, renal, and brown fat pads and observed the recombination product in multiple fat depots in cKO animals, but not CON (Fig. 5a). Fat tissue contains many cell types besides adipocytes, including endothelium and other vascular components, as well as hematopoietic cells such as macrophages. To examine *Osx-Cre* driven recombination in specific cells, we used confocal microscopy to examine fat pads from *Osx-Cre;TdTomato* (*TdT*) reporter mice raised in the same manner as the *IKKα* cKO mice. We found limited reporter expression in inguinal fat at 6 and 12 weeks of age, with substantially increased signal at 6 and especially 18 months (Fig. 5b). The pattern of TdT coincided with immunostaining for perilipin, demonstrating that recombination occurs in adipocytes. In contrast, we found no co-staining of TdT with CD45 (Fig. 5c), indicating that recombination in hematopoietic cells in fat is unlikely to be responsible for the observed low-fat phenotype. Therefore, we concluded that *Osx-Cre* drives recombination in peripheral adipocytes in an age-dependent manner.

### Aging *IKKα* cKO fat pads are smaller and show decreased leptin expression

**Figure 6:**
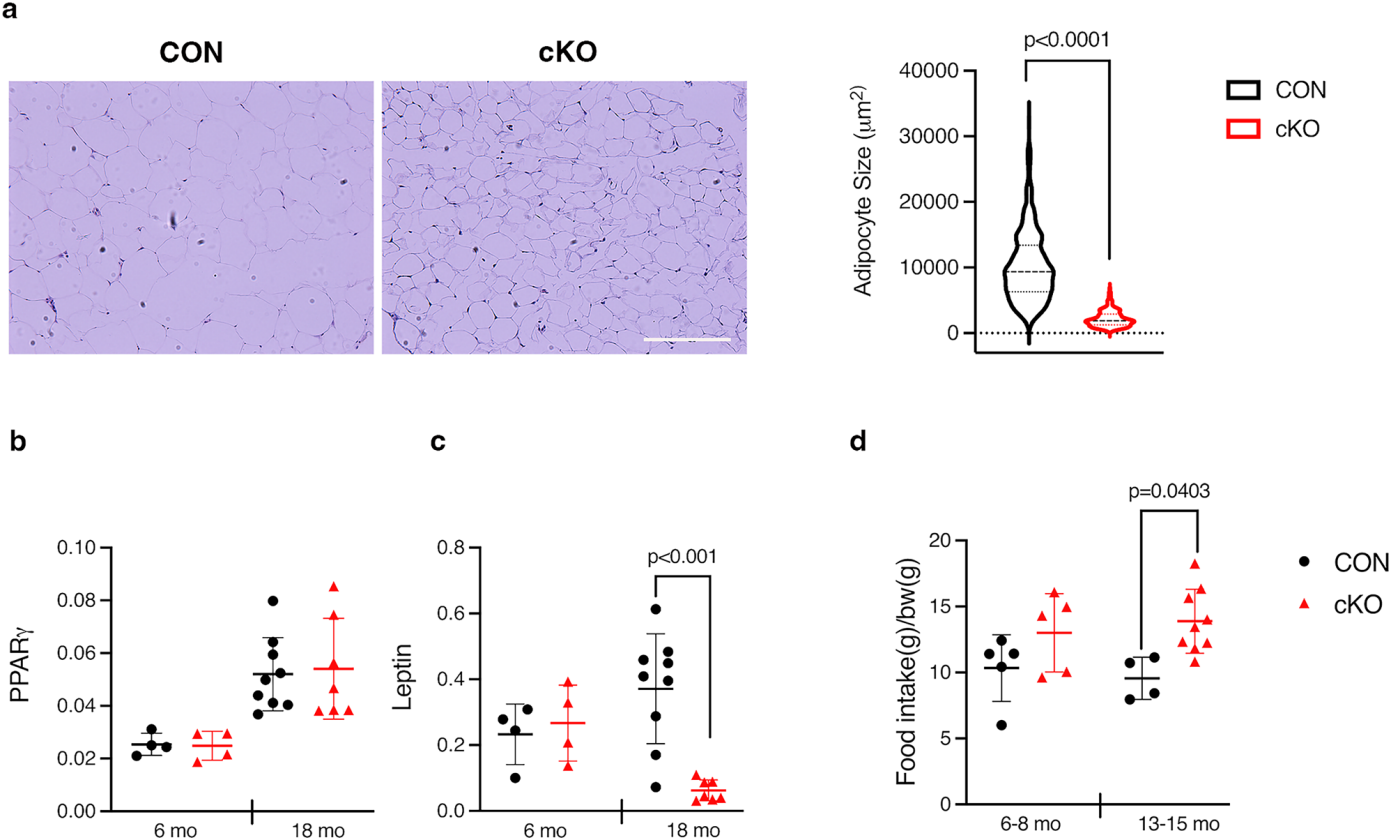
Aging *IKKα* cKO fat pads are smaller and show decreased leptin expression. (**a**) (*left*) Representative H&E staining of gonadal fat from 18 months old CON and cKO mice and (*right)* quantification of adipocyte size. CON = black, cKO = red. (**b**) qPCR for *PPARγ* from gonadal fat in CON (n = 4–9) and cKO (n = 4–7) mice at indicated ages. (**c**) qPCR for Leptin, as in (**b**). (**d**) Total food intake was measured daily over 3–4 days in CON (n = 4–5) and cKO (n = 5–9) mice at indicated ages and normalized to bodyweight. Data represented as mean ± SD. At least 350 adipocytes from 3 independent samples of each genotype were measured and unpaired, two-tailed student’s t-test was performed (for a). Technical duplicates were averaged (b,c), and 2-way ANOVA followed by Sidak multiple comparisons test was performed (b,c,d), with values indicated where significant.

To ascertain if there were any phenotypic differences between CON and cKO adipocytes that might be driving the loss of peripheral fat mass and improved glucose tolerance with age, we next measured adipocyte size and expression levels of various adipogenic markers. The average adipocyte area in gonadal fat pads from old cKO mice was substantially smaller compared to CON (Fig. 6a). qPCR analysis of fat pads between 6 and 18mo animals indicated that the key regulators of adipogenesis, PPARγ and C/EBPα^25^, were not different between genotypes with age (Fig. 6b and Fig S8a). Although adiponectin and the lipolysis enzymes (hormone sensitive lipase and adipose triglyceride lipase) trended modestly downward at both ages in cKO compared to CON animals, they did not reach statistical significance (Fig S8a, b). In contrast, with loss of IKKα, expression of leptin, the satiety hormone^26^, is markedly decreased in old cKO fat pads (Fig. 6c). Given the smaller size of cKO adipocytes and reduced leptin levels, we monitored food intake to see if loss of IKKα impacted feeding habits. Indeed, middle aged cKO animals showed increased food consumption relative to CON, and not a decrease that could drive fat loss (Fig. 6d). All together, this data suggests that cKO animals are protected from the perturbations to adipocyte function that occur during aging.

### *IKKα* cKO mice show a blunted response to high fat diet

**Figure 7:**
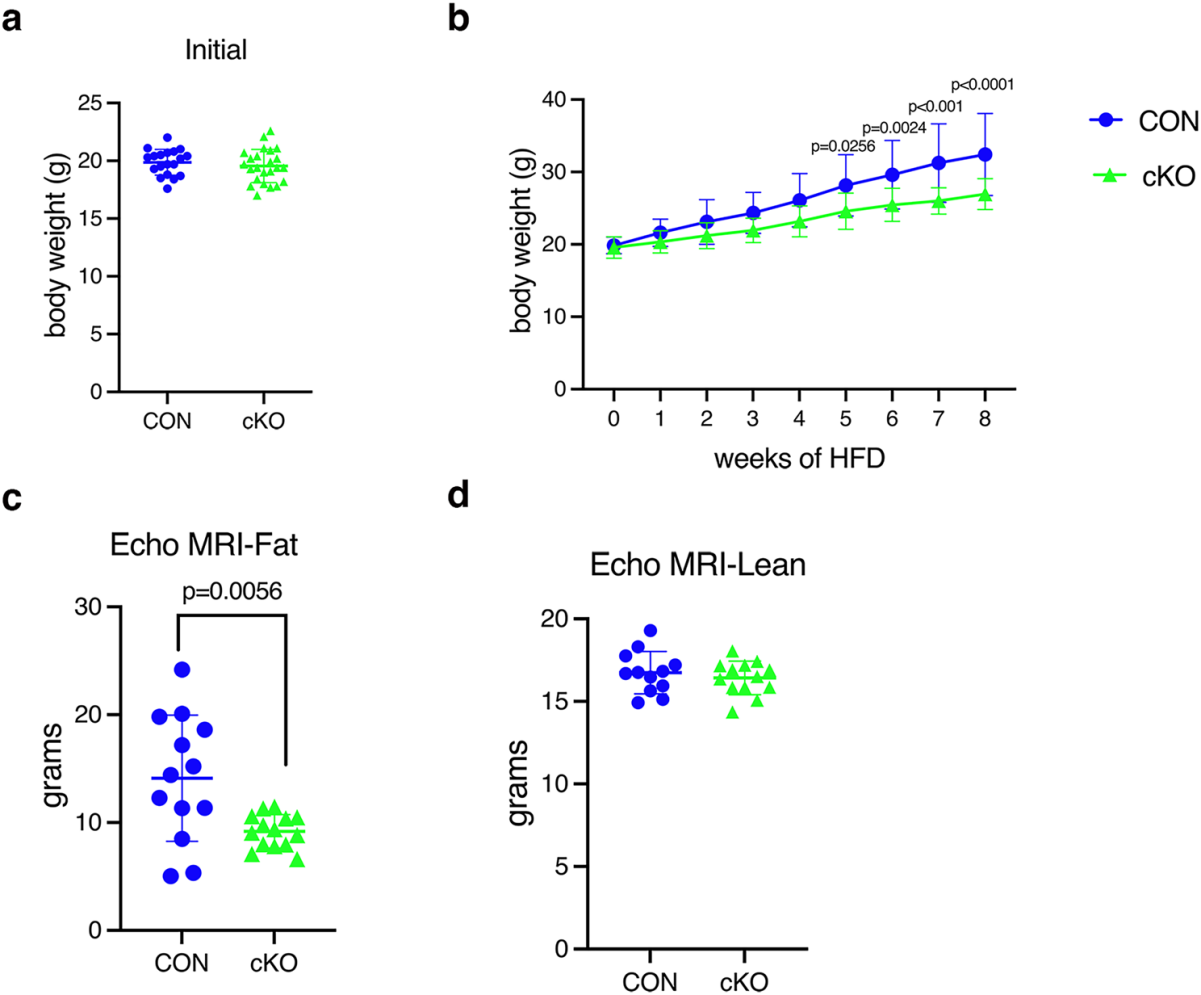
*IKKα* cKO mice show blunted weight and fat gain after HFD. Body weights (BW), (**a**) Initial or (**b**) after HFD for 8 wks. (**c**) EchoMRI of total fat mass or (**d**) total lean mass after 8wks on HFD. CON = blue, cKO = green. Data are represented as mean ± SD. Female mice (initial BW, n = 15–23; HFD BW, n = 12–18; EchoMRI, n = 10–14). Repeated measures 2-way ANOVA followed by Sidak multiple comparisons test for body weight on HFD for b and Student’s, unpaired, two-tailed t-test for a,c, and d. HFD = high fat diet.

To further investigate the fat phenotype observed in aging cKO mice, we fed younger animals according to a well-established high fat diet (HFD) paradigm as an alternative metabolic stress. Mice were maintained on this diet (60%kcal/fat) beginning at 8 weeks of age, prior to any difference in body weight between CON and cKO mice (Figs. 7a and S9a). Male cKO mice showed blunted weight gain which did not reach statistical significance following 8 weeks of HFD, although total fat mass was decreased by ECHO MRI (Fig S9b, c). Attenuation of weight gain in female cKO mice was significant (60% increase in CON vs 40% increase in cKO) (Fig. 7b), associated with a marked reduction in fat mass (Fig. 7c). Total lean mass was unchanged in both sexes, compared to CON (Fig. 7d and S9d).

**Figure 8:**
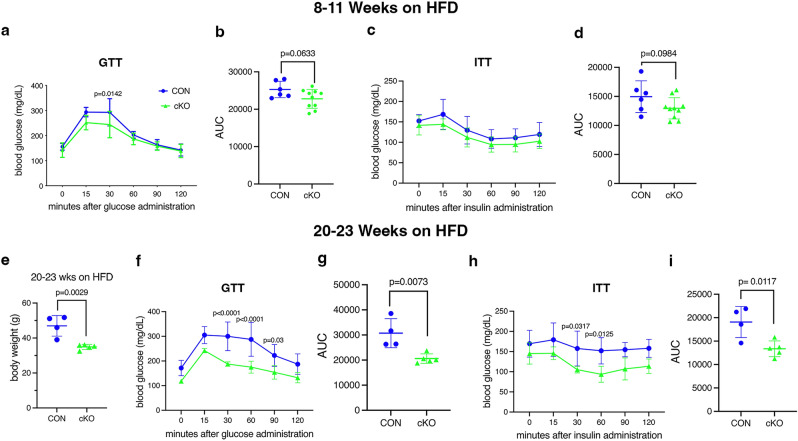
*IKKα* cKO mice show improved glucose metabolism after HFD. Blood glucose levels were measured during Glucose Tolerance Tests (GTT) and Insulin Tolerance Tests (ITT), which were initiated after a 6 h fast, in female mice. (**a**–**d**) Tests were performed after 8–11 wks on HFD (n = 6–10). (**a**) GTT, (**b**) area under the curve (AUC) for GTT, (**c**) ITT, and (**d**) AUC for ITT. (**e**–**i**) Tests were repeated on a subset of mice after 20–23 wks on HFD (n = 4–6). (**e**) body weight at time of GTT, (**f**) GTT, (**g**) AUC for GTT, (**h**) ITT, and (**i**) AUC for ITT. CON = blue, cKO = green. Data are represented as mean ± SD. Repeated measures 2-way ANOVA followed by Sidak multiple comparisons test for GTT and ITT or Student’s, unpaired, two-tailed t-test for body weight and AUC.

After 8–11 weeks on HFD, male cKO mice showed a trend towards better glucose tolerance, but this did not reach statistical significance in our cohorts (Fig S9e). Glucose tolerance in females after 8–11 weeks similarly trended better in cKO mice (Fig. 8a, b). Insulin tolerance was not significantly different in either sex at this point, although female cKO showed a slight trend towards better tolerance (Figs. 8c, d and S9f.). We then decided to continue HFD in a subset of females for 20–23 weeks. After this extended period, body weight continued to be lower in cKO, and both glucose and insulin tolerance were significantly improved compared to CON (Fig. 8e–i). Because significant changes in weight and fat mass occurred long before alterations in glucose metabolism, it is likely that the metabolic effects are secondary to, rather than the primary drivers of, weight gain.

## Discussion

In this study, we set out to examine the role of alternative NF-κB signaling in the osteolineage by targeting a key upstream kinase, IKKα, using *Osx-Cre*. Previously, we found that forced activation of this pathway using a constitutively active allele of NIK with the same *Osx-Cre* driver enhanced both basal and stimulated bone formation^13^. Here, male cKO mice showed no differences in bone mass up to 18 months of age, and mechanical loading by tibial compression failed to generate any differences in bone formation. Female mice also had normal bone mass at 4 months of age. Thus, under basal and non-inflammatory loading conditions, IKKα does not seem to have a clear role, either positive or negative, in bone formation. One limitation of our bone analysis is that we did not follow females over time, despite our finding that global loss of alternative NF-κB components NIK and RelB has greater effect in females. However, in those models, the differences are clear by 10 weeks of age^12^. It is possible that IKKα may impact bone formation in the context of strong inflammatory stimuli such as inflammatory arthritis models, which were not examined here.

Surprisingly, while we followed cKO mice for a potential age-dependent effect in bone, we were struck by overt differences in weight that widened with age. Further investigation revealed that this was driven primarily by lower fat mass, which preceded changes in glucose metabolism and modest changes in lean mass. Adipocytes in old cKO fat pads had a much smaller average area than CON. Expression of early and mature adipocyte markers was normal or trended modestly lower in cKO fat pads from both young and aged mice. In contrast, leptin was dramatically decreased with age. Indeed, cKO animals displayed a trend towards increased food intake that became significant by middle age, when weights were distinctly lower. Thus, decreased appetite is unlikely to be driving the loss of fat. Using HFD as a metabolic stress in young cKO mice, we also observed less fat accumulation. However, additional studies are necessary to determine if this is driven by similar mechanisms as in aging.

Given the striking loss of fat in aging cKO mice in the absence of any changes in bone, we considered the possibility that recombination outside of bone was responsible for the phenotype. PCR of genomic DNA from peripheral fat depots in middle aged mice showed recombination of the *IKKα* allele, albeit less than in bone. Examination of inguinal fat pads from *Osx-Cre;TdT* reporter mice showed robust TdT expression only in adipocytes from aged mice. Despite identification of TdT in several subsets of CD45 + cells in a previous study using the same line of reporter mice^27^, we did not identify any such cells in the inguinal fat sections at any age. Strengthening our assertion that *Osx-Cre* drives loss of IKKα in adipocytes, expression of endogenous *Osx* has been reported during differentiation of the preadipocytic cell line 3T3-L1^28^. Furthermore, the *Osx-Cre* allele has established recombination activity in many extraskeletal tissues including synovium^29^, intestinal epithelium^30,31^, and kidney^32^, as well as in some hematopoietic stem and progenitor cells^27^. In tumor bearing mice, *Osterix*, as well as the *Osx-Cre* allele, is also expressed in a subset of cancer-associated fibroblasts with a dual fibroblast/osteogenic signature^27^. Recently, we found subcutaneous sarcomas, but not bone tumors, in mice expressing a transgene driven by *Osterix-Cre*^33^. Thus, it is possible that loss of IKKα in adipocytes themselves is responsible for the observed fat phenotype.

Most studies of the role of NF-κB in adipocytes have focused on IKKβ, an apex kinase in the canonical pathway. Manipulation of IKKβ in adipocyte lineage cells in mouse models has complex and stage-specific effects on fat mass, glucose homeostasis, and response to HFD^34–37^. Thus, the role of IKKβ in the adipocyte lineage is complex, and the effects of aging have not been reported in any of these models. Data on the role of IKKα or other components of alternative NF-κB in metabolism is even more limited, with roles described in pancreatic islets, hepatocytes, and skeletal muscle^9^. In particular, HFD feeding hyperactivated alternative NF-κB in pancreatic islets resulting in β-cell failure^38^. While it is possible that age or HFD activates *Osx-Cre* in a metabolic organ such as pancreas, we find changes in weight prior to changes in glucose metabolism, suggesting that this is a secondary response, rather than a driver of the cKO phenotype. Future studies using an adipocyte-specific *Cre* such as *Adiponectin-Cre* are needed to determine if there is a cell-autonomous role for IKKα in adipocytes.

In addition to the unexpected finding of a fat phenotype driven by *Osx-Cre*, we also did not anticipate the strong effect of age on activation of this *Cre*. Previous studies utilizing this *Cre* driver primarily utilize mice under 6 months of age, and the few studies with mice at or beyond 1 year of age did not describe, or specifically look for, Cre expression outside of bone^39–41^. Since this study was undertaken to examine the role of IKKα in bone, studies of fat and metabolism were not initially planned. By the time the fat phenotype in aging males was discovered, it was not practical to undertake a similar study in females. Therefore, we decided to investigate whether differences in fat could be accelerated using HFD in both sexes. Interestingly, although both male and female cKO mice had less fat than CON after 8 weeks on the obesogenic diet, the difference in overall body weight was more pronounced in females. Thus, like osteoclasts^12^, adipocytes may have differential sensitivity to the alternative NF-κB pathway by sex. Alternatively, HFD may induce *Osx-Cre* expression in adipocytes, similar to aging, and the rate of this may differ between males and females.

In sum, using an *Osx-Cre* driven conditional knockout approach, we found no clear role for IKKα in the osteolineage in either basal or mechanically stimulated conditions, but rather an intriguing role for IKKα in fat accumulation. cKO mice were protected from age-related or HFD-induced metabolic dysfunction, and remained lean. With increasing recognition of bone as an endocrine organ, it is tempting to conclude that phenotypes arising from conditional alleles driven by *Osx-Cre* are due to the osteolineage. However, our finding of an age-related increase in *Osx-Cre* expression in peripheral fat indicates that expression outside of bone should be considered when metabolic phenotypes are identified in aged animals. More comprehensive analysis of conditional mouse models with sensitive reporters like TdT is likely to uncover other so-called off-target effects that arise with aging in many *Cre* lines. Although it is not yet clear if the adipocyte is the target cell, the results shown here suggest that inhibition of IKKα, or potentially other alternative NF-κB pathway components, may reduce fat accumulation with age and preserve glucose metabolism.

## Materials and methods

### Mice

Mice were communally housed in a pathogen-free barrier facility, with controlled temperature and 12-h light/dark cycles. They had ad libitum access to fresh water and standard rodent chow (5058; Purina, St. Louis, MO, USA) unless otherwise indicated. Protocols were approved by Institutional Animal Studies Committee at Washington University School of Medicine (ASC protocols 20170025 and 19-1059) and all methods were performed in accordance with the relevant guidelines and regulations, including with ARRIVE guidelines.

The *IKKα flox* transgenic line was generated as described elsewhere^42^. *Osx1-GFP::Cre* mice (006361; The Jackson laboratory, ME USA) express *Cre-recombinase* under control of a Tet-OFF cassette^23^. The *IKKα flox* transgenic and *Osx1-GFP:Cre (Osx-Cre)* parental mouse lines were maintained separately due to strain differences (C57Bl/6J and mixed C57Bl/6J and CD1, respectively). *Osx-Cre;IKKα*^*fl/fl*^ (cKO) mice and *Cre-negative WT;IKKα*^*fl/fl*^ (CON) littermates were maintained on a 200 ppm doxycycline chow diet (1816332–203; Purina Test Diet, St. Louis, MO, USA). Pups were switched to standard rodent chow at weaning (P21-P22). To generate *TdTomato (TdT)* reporter mice, *Osx-Cr*e mice were crossed with *TdT* mice (007909; The Jackson Laboratory, ME USA), and pups were maintained on the same doxycycline chow until weaning (P21-P22). Age and sex-matched animals from the same colony were used in all experiments.

### Micro-computed tomography

The right tibia of mice was scanned by microCT in vivo (VivaCT 40, Scanco, Brüttisellen, Switzerland) at 10.5 mm resolution (70 kVp, 114 mA, 8 W, 100 ms integration time). Cancellous bone parameters were measured at a 1 mm region distal to the end of the tibial growth plate. Cortical measurements were made at the tibial mid-shaft (1 mm region defined 5 mm proximal to the distal tibiofibular junction). Bone indices are reported in accordance with established standards^43^.

### Dual-energy X-ray absorptiometry (DXA)

Whole body DXA scans were performed on 18 month male CON and cKO mice using a Faxitron UltraFocus 100 machine (Faxitron, Buffalo Grove, IL, USA).

### Mechanical loading and dynamic histomorphometry

Unilateral axial tibial compression (Electropulse 1000; Instron, Norwood, MA, USA) and dynamic histomorphometry (Bioquant Osteo software v18.2.6; Bioquant Image Analysis Corp., Nashville, TN, USA) was performed on the right tibiae of 16-week-old, male mice as previously described^13^. The left tibia served as the contralateral non-loaded control. Strain gauging was performed to determine the force necessary for a 2000 microstrain deformation (9.6 N, both genotypes). In samples where the mineral apposition rate was zero, an imputed value of 0.1 was used to allow for statistical comparisons. All measurements were acquired in a blinded fashion and reported in accordance with published standards^43^.

### Genomic DNA recombination

Long bones were flushed of marrow and crushed in TRIzol (15596026; Invitrogen, USA) using a Navy RINO lysis kit and Bullet Blender Tissue Homogenizer (Next Advance, Troy, NY, USA). Peripheral fat depots were processed similarly to crushed bone samples. Cell cultures were rinsed 2 × with PBS, then lysed directly in TRIzol. Genomic DNA was extracted using a back extraction buffer (4 M guanidine thiocyanate, 50 mM sodium citrate, 1 M Tris) and alcohol precipitation. 50 ng of input gDNA per sample using GoTaq polymerase (M7123; Promega, USA). PCR cycling conditions: 94 °C–4 min; (94 °C–30 s, 55 °C–45 s, 72 °C–1.5 min) × 30 cycles; 72 °C–10 min; 12 °C hold. Primer sequences for recombination: Forward—CTT TGC CAT CAT CTC TCC GGT TTG TAA; Reverse—CAA TAG GAT AAT CAC TAA GCA CAG T.

### High fat diet

Mice were fed a 60% kcal/fat diet (D12492; Research Diets Inc, New Brunswick, NJ, USA), ad libitum, beginning at 8–9 weeks of age. Body weight was measured once a week. At sacrifice, peripheral fat pads were weighed after removal of any gross contaminating tissue.

### EchoMRI

Lean and fat mass was assessed by EchoMRI body composition analysis (EchoMRI LLC, Houston TX, USA) in non-fasted mice. Each mouse was scanned twice and the average value was used for analysis.

### Glucose and insulin tolerance tests

Mice were housed on aspen bedding and administered intraperitoneally a 1 mg/g dose of dextrose or 0.75U/kg of insulin (HumulinR U-100; Eli Lily, USA) after a 6 h or overnight fast (food only), as indicated. Tail vein blood was sampled at intervals over a 2 h period and blood glucose was measured using a Bayer Contour meter (9556C; Bayer HealthCare, Mishawaka, IN, USA) and accompanying test strips.

### Immunostaining and fluorescence imaging

Inguinal fat was embedded in optimal cutting temperature compound (OCT; 23-730-571, Thermo Fisher Scientific, USA) and flash frozen at − 80 °C. Embedded tissues were post fixed in 10% neutral buffered formalin and cut at 50 μm on a cryostat (Leica, Buffalo Grove, IL, USA). For immunostaining, cut sections on glass slides were blocked in 10% goat serum in Tris- NaCl- Tween (TNT) buffer before incubation for 24 h with primary antibodies; anti-perilipin 1 (N-terminus) guinea pig polyclonal (LS-C665927-100,GP29; LS Bio, USA) (1:1000), anti-CD45 monoclonal antibody (30-F11)(14-051-82; Thermo Fisher Scientific, USA) (1:200) overnight at 4 °C. After washing 3 × 5 min with TNT, secondary antibodies (Alexa 488 (A-11073; Thermo Fisher Scientific, USA) or Alexa 488 (A-48269; Thermo Fisher Scientific, USA) in TNT buffer were applied for 1 h at room temperature. The sections were then washed 3 × 5 min in TNT buffer, before mounting with Fluoromount-G (00-4958-02; Thermo Fisher Scientific, USA). Images were captured using Leica DMi8 automated inverted microscope equipped with ACS APO 20x/0.60 Lens (Leica Microsystems).

### Adipocyte size

Gonadal fat pads were excised and fixed in 10% neutral buffered formalin. 5 μm paraffin-embedded sections were subjected to hematoxylin and eosin staining. Standard bright field images were acquired. Adipocyte area was measured using Image J (NIH, Version 1.53).

### Quantitative real-time PCR

Peripheral fat pads were homogenized in TRIzol as described above. Total RNA was extracted with chloroform:isoamyl alcohol. An equal volume of 70% ethanol was added followed by purification with a NucleoSpin RNA II kit (740955.50; Takara Bio, USA). cDNA was reverse-transcribed using 1 μg of total RNA per manufacturer’s instructions (639549; Takara Bio, USA). PCR cycling was performed using iTaq Universal SYBR Green Supermix (1725121; Bio-Rad, USA) on an Applied Biosystems QuantStudio 3 using the following settings: 50 °C for 2 min, 95 °C for 10 min, and then 40 cycles of 95 °C for 15 s and 60 °C for 1 min. Relative expression was calculated as 2^− (target CT − GAPDH CT)^. Primer sequences are listed in Supp Table 2.

### Food intake studies

Mice were acclimated to single housing and then food intake was measured daily for 3–4 days by subtracting starting and ending normal chow pellet weights. Total food intake was averaged per mouse and expressed normalized to body weight.

### Statistical analysis

All statistics were computed using GraphPad Prism software (Version 9.2.0, GraphPad Software, Inc., La Jolla, CA, USA). Values of p < 0.05 were considered significant and data are presented as mean ± SD. For pairwise comparisons, either a student’s, unpaired, two-tailed t-test or student’s paired, two-tailed t-test was used. A Welch’s correction was applied to the t-test if the F-test to compare variances was significantly different. For multiple group comparisons, a 2-way ANOVA followed by Tukey’s multiple comparisons test was performed. For repeated measure group comparisons, a 2-way repeated measures ANOVA followed by Sidak’s multiple comparisons test was performed. A mixed-effect model was used if sample sizes differed. Specific statistical tests and sample sizes are indicated in the respective figure legends.

## Supplementary Information

Supplementary Information.
