# Supplementary material for: Conditional loss of IKKα in Osterix + cells has no effect on bone but leads to age-related loss of peripheral fat
Source: Sci Rep. 2022 Mar 22;12:4915. doi: 10.1038/s41598-022-08914-6 (PMC8940989; doi:10.1038/s41598-022-08914-6)

Supplementary Material

Conditional loss of IKKα in Osterix+ cells has no effect on bone but leads to age-related loss of peripheral fat

Jennifer L Davis, Nitin Kumar Pokhrel, Linda Cox, Nidhi Rohatgi, Roberta Faccio, and Deborah J Veis

Supplementary Methods:

Osteoblast culture

Whole bone marrow was flushed from the long bones and bone marrow stromal cells (BMSCs) were expanded on tissue culture plates in growth media until subconfluent (7-10 days). Cells were replated into 6-well tissue culture plates (1e6 cells) in either growth media alone or standard osteogenic media (alpha-MEM, no ascorbic acid (Gibco # A1049001), 10% FBS, 2mM L-glutamine, Penicillin/Streptomycin, 50ug/mL ascorbic acid (Sigma A8960) and 10mM β-glycerol phosphate (VWR #101171-158)) for desired time points during osteoblast differentiation. Media was changed every 3 days.

Alkaline Phosphatase/Alizarin Red (ALP/AR) staining

Osteoblast culture was performed as described above. Alkaline phosphatase staining was visualized using a leukocyte alkaline phosphatase kit (Sigma #85L-2). Briefly, cells were rinsed 2x with PBS, fixed for 30 seconds in citrate buffered acetone, rinsed 2x with water, then stained with ALP solution for 40 minutes (fast blue RR salt plus Naphthol As-Mx Phosphate Alkaline Phosphate Solution 0.25). For dual ALP/AR staining, cells were first stained with ALP and then stained with 0.4% Alizarin Red-S in water (Sigma #A-5533) for 10 minutes.

Ex-vivo Micro CT

The left tibia was scanned ex-vivo (µCT40, Scanco, Brüttisellen, Switzerland) at 16 weeks of age (10 µm resolution, 55 kVp, 72µA, 4W, 300ms integration time). The region of interest was defined at 1 mm distal to the end of the tibial growth plate.

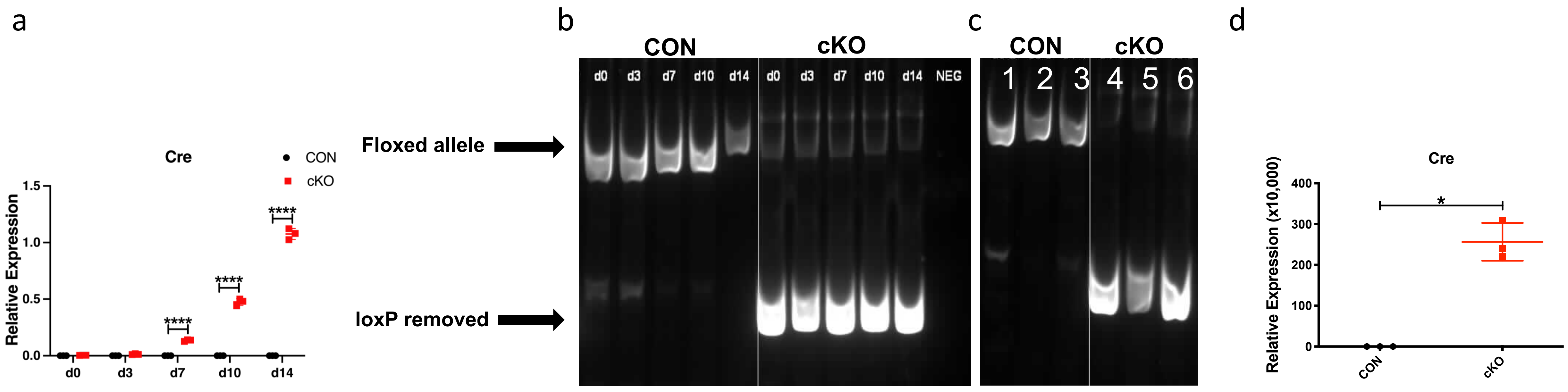

Supplementary Figure 1: Recombination of the IKKα floxed allele and Cre mRNA expression in both osteogenic culture and crushed bone from CKO mice. BMSCs were treated with standard osteogenic media containing β-glycerol phosphate and ascorbic acid for 0-14 days. CON = black, cKO = red. **a)** qPCR for Cre expression in OB cultures. **b)** PCR of genomic DNA from CON or cKO cultures over the course of differentiation showing presence of the recombination product (~460bp) only in cKO samples. NEG = negative control lane, primers only. **c)** genomic DNA PCR of flushed, crushed bone. Male mice, age 12wks. **d)** qPCR for Cre expression in flushed, crushed bone. Data are represented as mean ± SD. Representative data shown for n=3 independent experiments for a,b. n=3 biological replicates for c,d. 2-way ANOVA followed by Tukey's multiple comparison test (a) or Student's, unpaired, two-tailed t-test (d): **p<0.05,* ****p<0.0001.

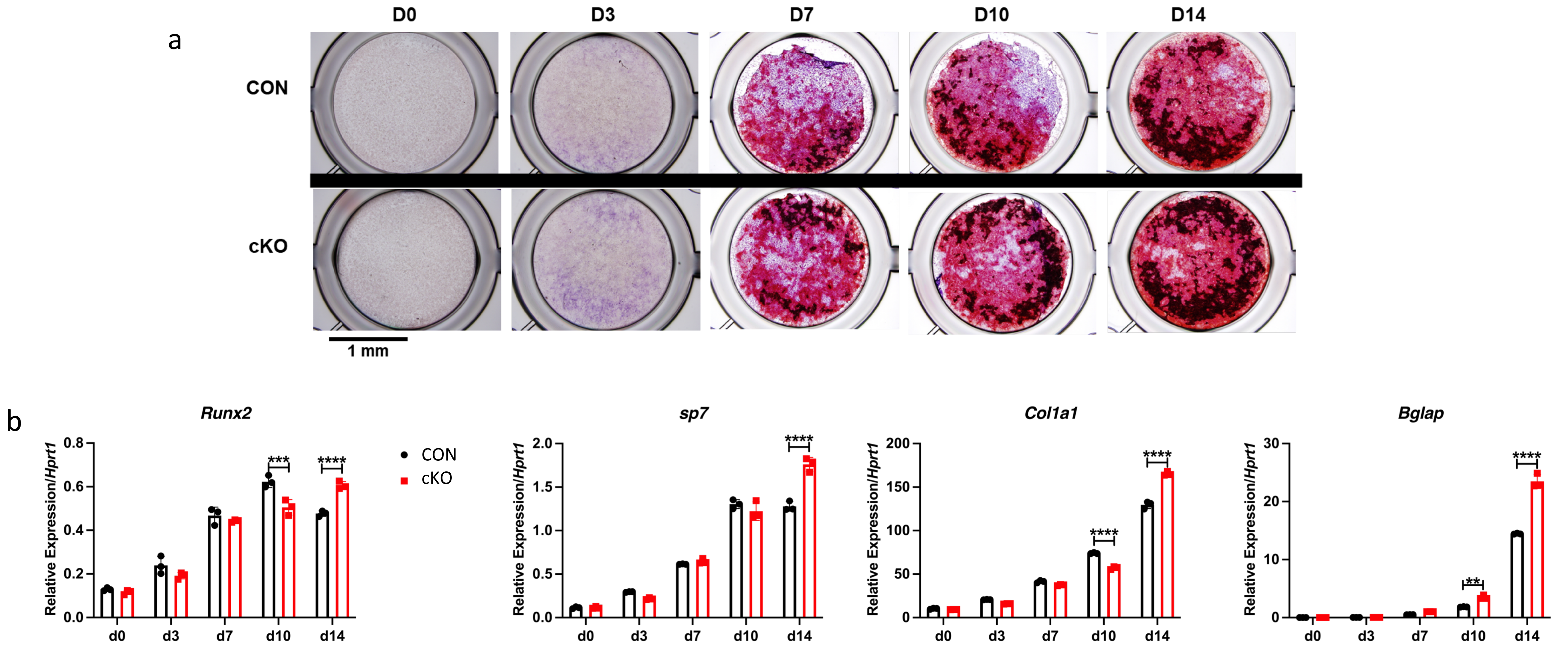

Supplementary Figure 2. IKKα cKO osteogenic cultures show upregulated osteoblast differentiation. BMSCs were treated with standard osteogenic media containing β-glycerol phosphate and ascorbic acid for the indicated time period. **a)** Representative images of ALP and AR staining of CON and cKO cultures over the course of OB differentiation. **b)** qPCR showing relative expression of multiple OB markers. CON = black, cKO = red. Data are represented as mean ± SD. Representative of 3 independent pairs of biological replicates. 2-way ANOVA followed by Tukey's multiple comparison test: *$p<0.05$, **$p<0.01$, ***$p<0.001$, ****$p<0.0001$.

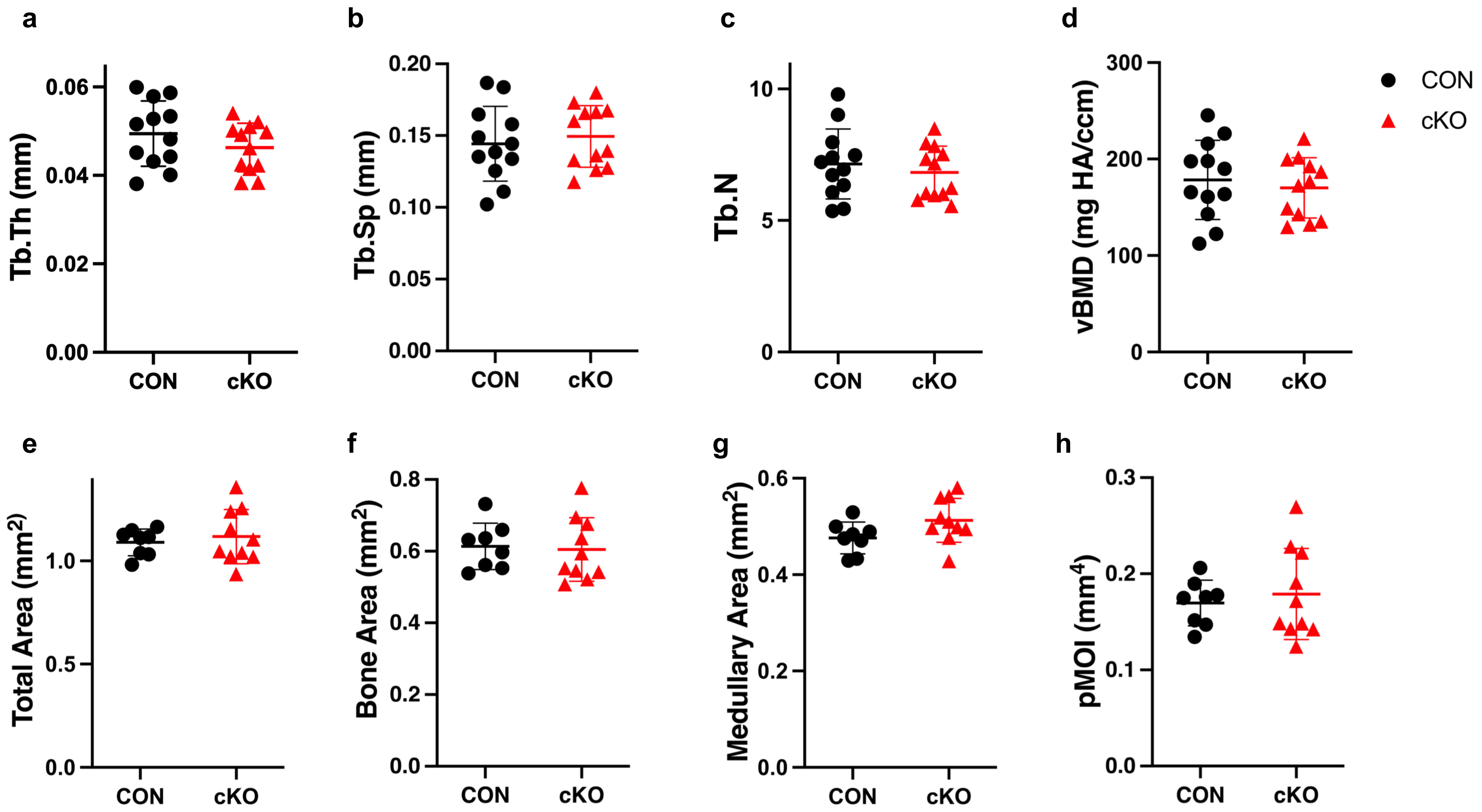

Supplementary Figure 3. 6 week old IKKα cKO mice display similar basal bone mass to CON.

In vivo microCT measurements of the cancellous **(a-d)** and cortical **(e-h)** compartments in the tibia of male 6 wk old mice. **a)** trabecular thickness (Tb.Th), **b)** trabecular spacing (Tb.Sp), **c)** trabecular number (Tb.N), **d)** volumetric bone mineral density (vBMD), **e)** total area, **f)** bone area, **g)** medullary area, **h)** polar moment of inertia (pMOI). Data are represented as mean ± SD. n=12 for a; n=8-10 for b. All non-significant by student's, unpaired, two-tailed t-test.

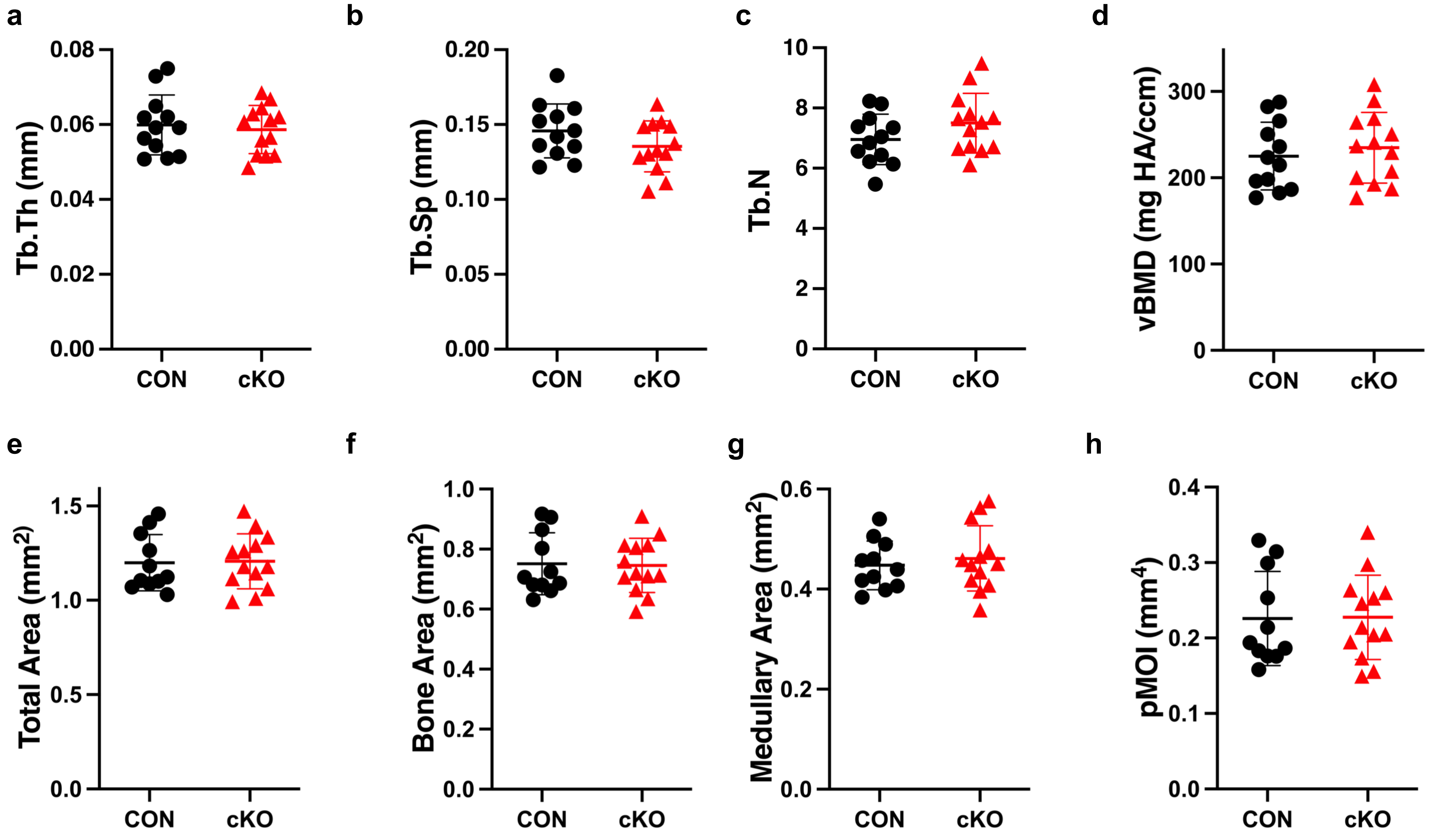

Supplementary Figure 4. 12 week old IKKα cKO mice have normal bone mass compared to CON. *In vivo* microCT measurements of the cancellous **(a-d)** and cortical **(e-h)** compartments in the tibia of male 12 wk old mice. **a)** trabecular thickness (Tb.Th), **b)** trabecular spacing (Tb.Sp), **c)** trabecular number (Tb.N), **d)** volumetric bone mineral density (vBMD), **e)** total area, **f)** bone area, **g)** medullary area, **h)** polar moment of inertia (pMOI). Data are represented as mean ± SD. n=11-13. All non-significant by student's, unpaired, two-tailed t-test.

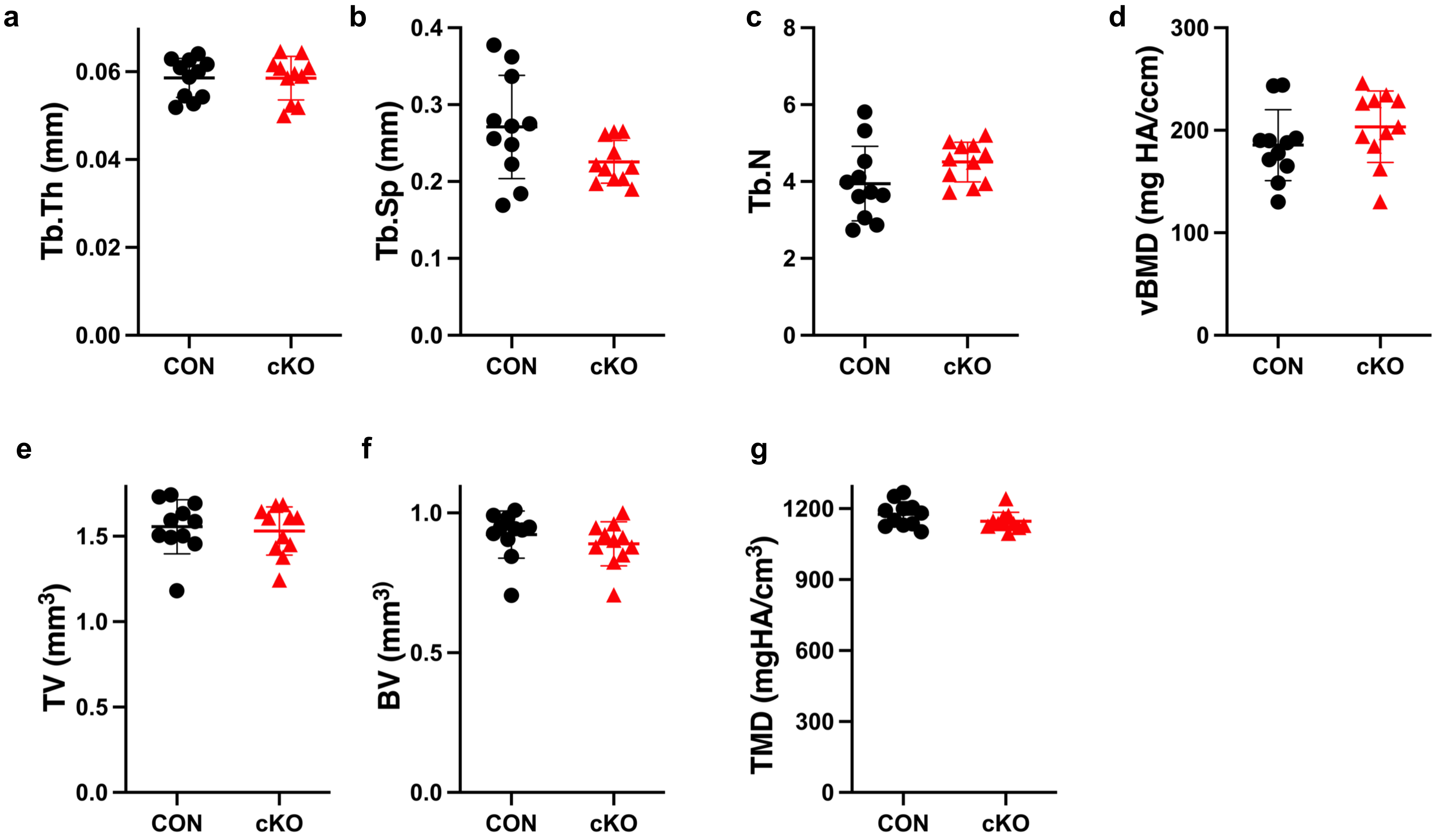

Supplementary Figure 5. 6 month old IKKα cKO mice have comparable bone mass to CON. *In vivo* microCT measurements of the cancellous **(a-d)** and cortical **(e-g)** compartments in the tibia of male 6 mo mice. **a)** trabecular thickness (Tb.Th), **b)** trabecular spacing (Tb.Sp), **c)** trabecular number (Tb.N), **d)** volumetric bone mineral density (vBMD), **e)** total volume (TV), **f)** bone volume (BV), **g)** tissue mineral density (TMD). Data are represented as mean ± SD. n=11, each genotype. All non-significant by Student's, unpaired, two-tailed t-test.

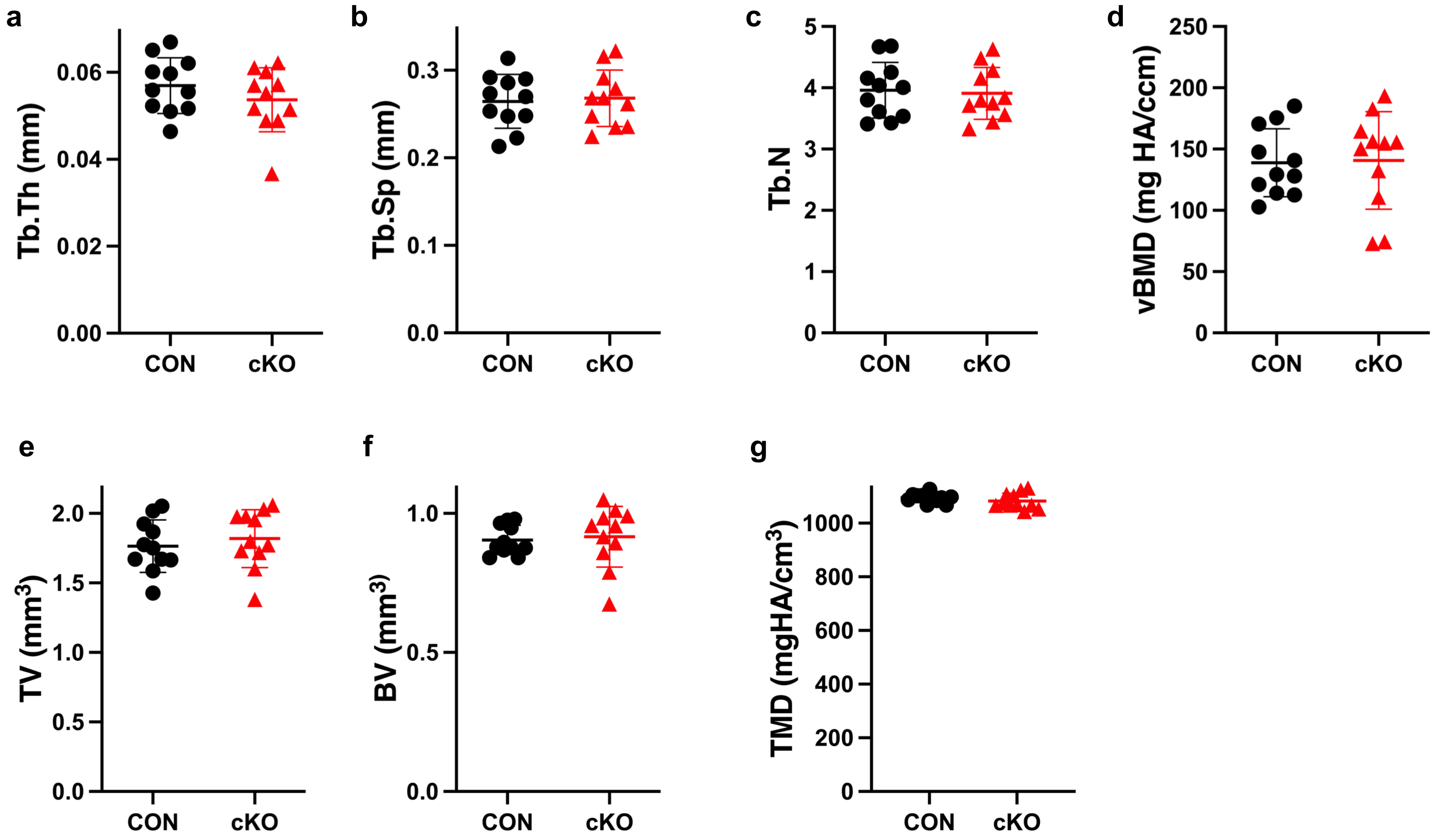

Supplementary Figure 6. 12 month old IKKα cKO mice have bone mass indistinguishable from CON. *In vivo* microCT measurements of the cancellous **(a-d)** and cortical **(e-g)** compartments in the tibia of male 12 mo mice. **a)** trabecular thickness (Tb.Th), **b)** trabecular spacing (Tb.Sp), **c)** trabecular number (Tb.N), **d)** volumetric bone mineral density (vBMD), **e)** total volume (TV), **f)** bone volume (BV), **g)** tissue mineral density (TMD). Data are represented as mean ± SD. n=11, each genotype. All non-significant by Student's, unpaired, two-tailed t-test.

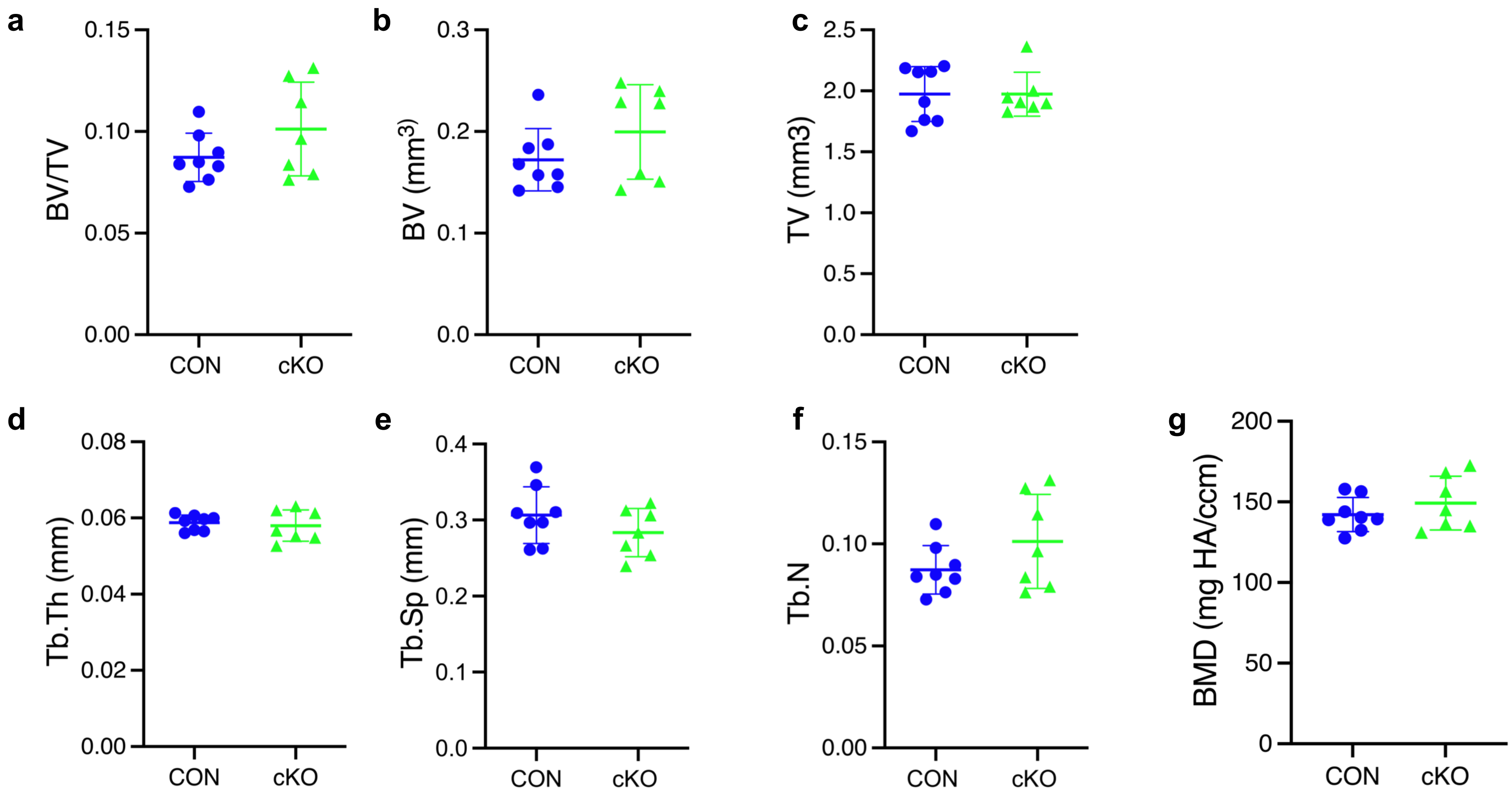

Supplementary Figure 7: 16 week old female IKKα cKO mice have similar bone volume to CON mice.

Ex vivo microCT measurements of the cancellous compartment in 16 wks female mice. **a)** BV/TV, **b)** BV, **c)** TV, **d)** trabecular thickness (Tb.Th), **e)** trabecular spacing (Tb.Sp), **f)** trabecular number (Tb.N), **g)** volumetric bone mineral density (vBMD), . **c)** BMD. Data are represented as mean ± SD. n=7-8, each genotype. All non-significant by Student's, unpaired, two-tailed t-test.

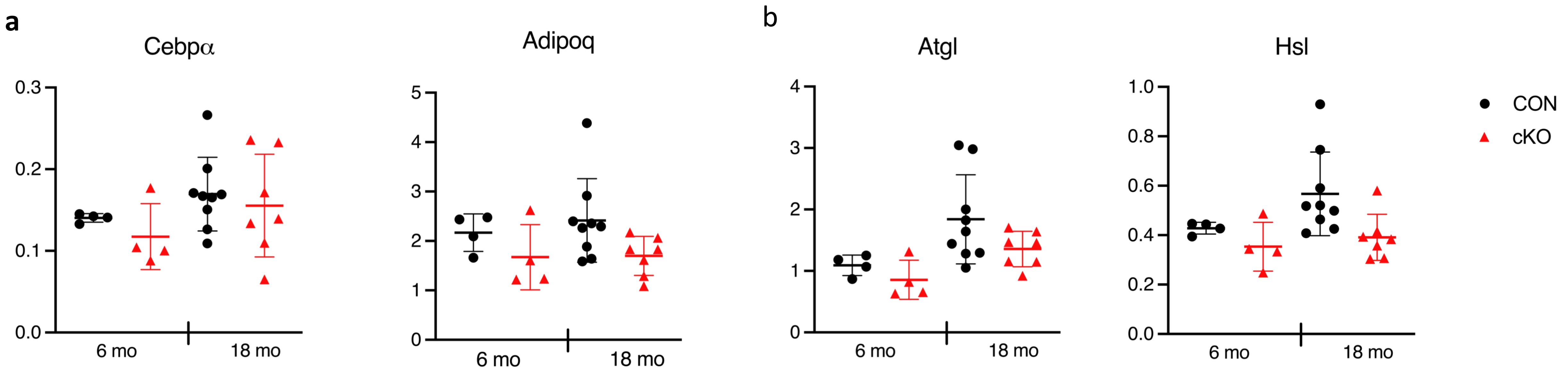

Supplementary Figure 8: Adipocyte marker expression in young adult and aged fat pads.
mRNA was isolated from gonadal fat pads at the indicated ages, and qPCR was performed for **a)** adipogenic markers Cepbα and Adipoq, and **b)** lipolysis markers Atgl and Hsl in CON (n=4-9) and cKO (n=4-7) mice. Data represented as mean ± SD. CON=black, cKO=red. Technical duplicates were averaged, and 2-way ANOVA followed by Sidak multiple comparisons test was performed. Although all show a trend toward decrease in cKO, no changes were significant ($p<0.05$).

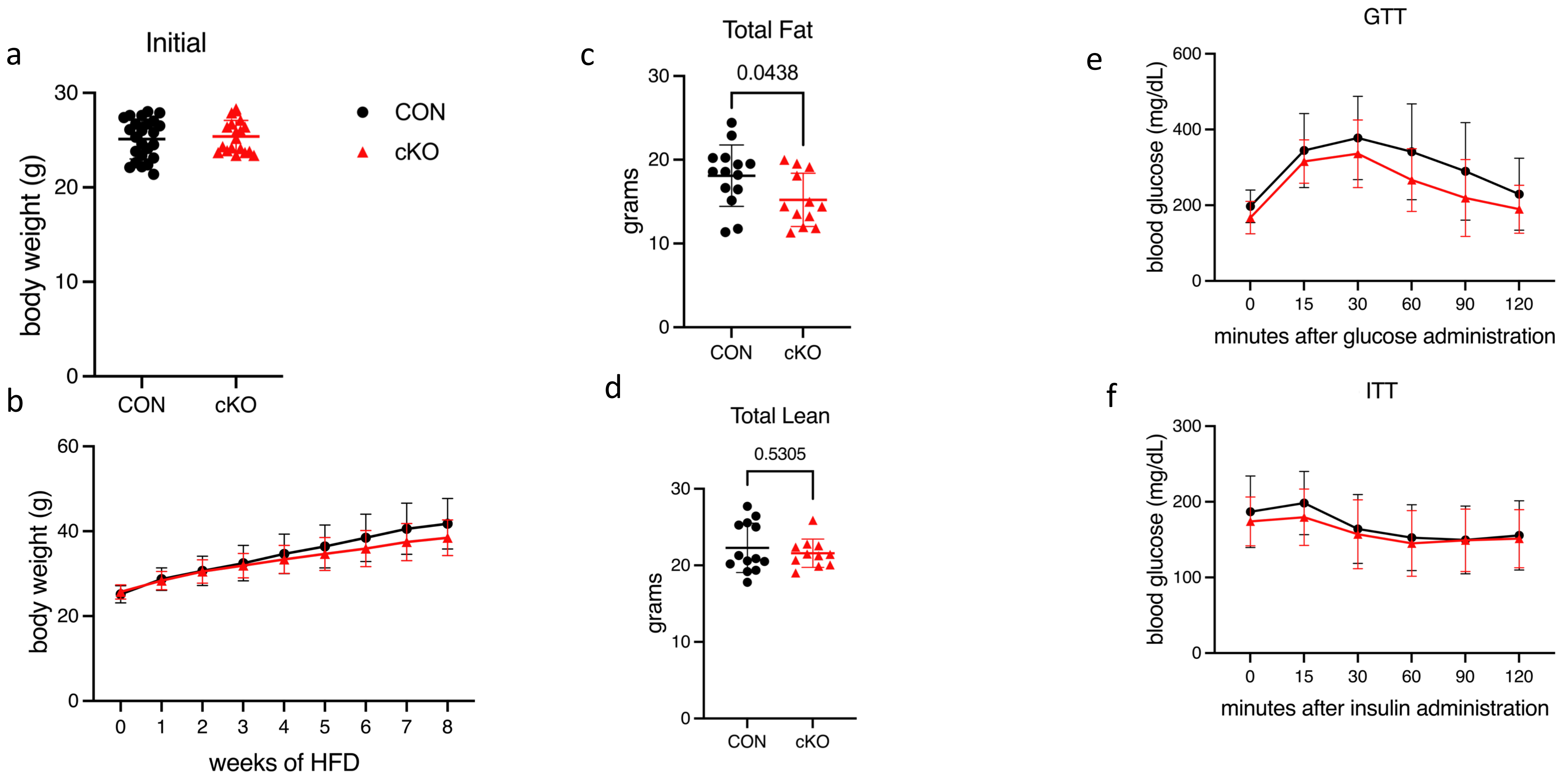

Supplementary Figure 9: Metabolic phenotype of male CON and IKKα cKO mice after 8 weeks on HFD. Body weight **a)** Initial or **b)** after 8 wks HFD (n=16-19). EchoMRI analysis after 8 wks HFD **c)** total fat mass or **d)** total lean mass (n=11-14). Blood glucose measurements after 8 wks HFD during **e)** GTT and **f)** ITT (n=10). Data are represented as mean ± SD. Repeated measures 2-way ANOVA followed by Sidak multiple comparisons test for body weight, GTT, and ITT or Student's, unpaired, two-tailed t-test for EchoMRI. Non-significant between genotypes except for Total Fat. HFD = high fat diet.

Supplementary Table 1: Biorad qPCR primers

Gene	Assay ID/Primer
**Cola1**	qMmuCID0021007
**Bglap**	qMmuCED0041364
**Runx2**	qMmuCED0049270
**Sp7**	qMmuCED0039982
**IKKa**	qMmuCID0005344
**Hprt1**	qMmuCED0045738

Supplementary Table 2: qPCR primers

Gene	Primer
**Cebpα**	F-GACATCAGCGCCTACATCGA
	R-TCGGCTGTGCTGGAAGAG
**PPARγ**	F-ACCACTCGCATTCCTTTGAC
	R-TGGGTCAGCTCTTGTGAATGA
**Adipoq**	F-GCACTGGCAAGTTCTACTGCAA
	R-GTAGGTGAAGAGAACGGCCTTGT
**Atgl**	F-TGTGGCCTCATTCCTCCTAC
	R-TCGTGGATGTTGGTGGAGCT
**Hsl**	F-GCTGGGCTGTCAAGCACTGT
	R-GTAACTGGGTAGGCTGCCAT
**Leptin**	F-GAGACCCCTGTGTCGGTTC
	R-CTGCGTGTGTGAAATGTCATTG
**Gapdh**	F- AGGTCGGTGTGAACGGATTTG
	R- TGTAGACCATGTAGTTGAGGTCA

Uncut gel for Fig 5a

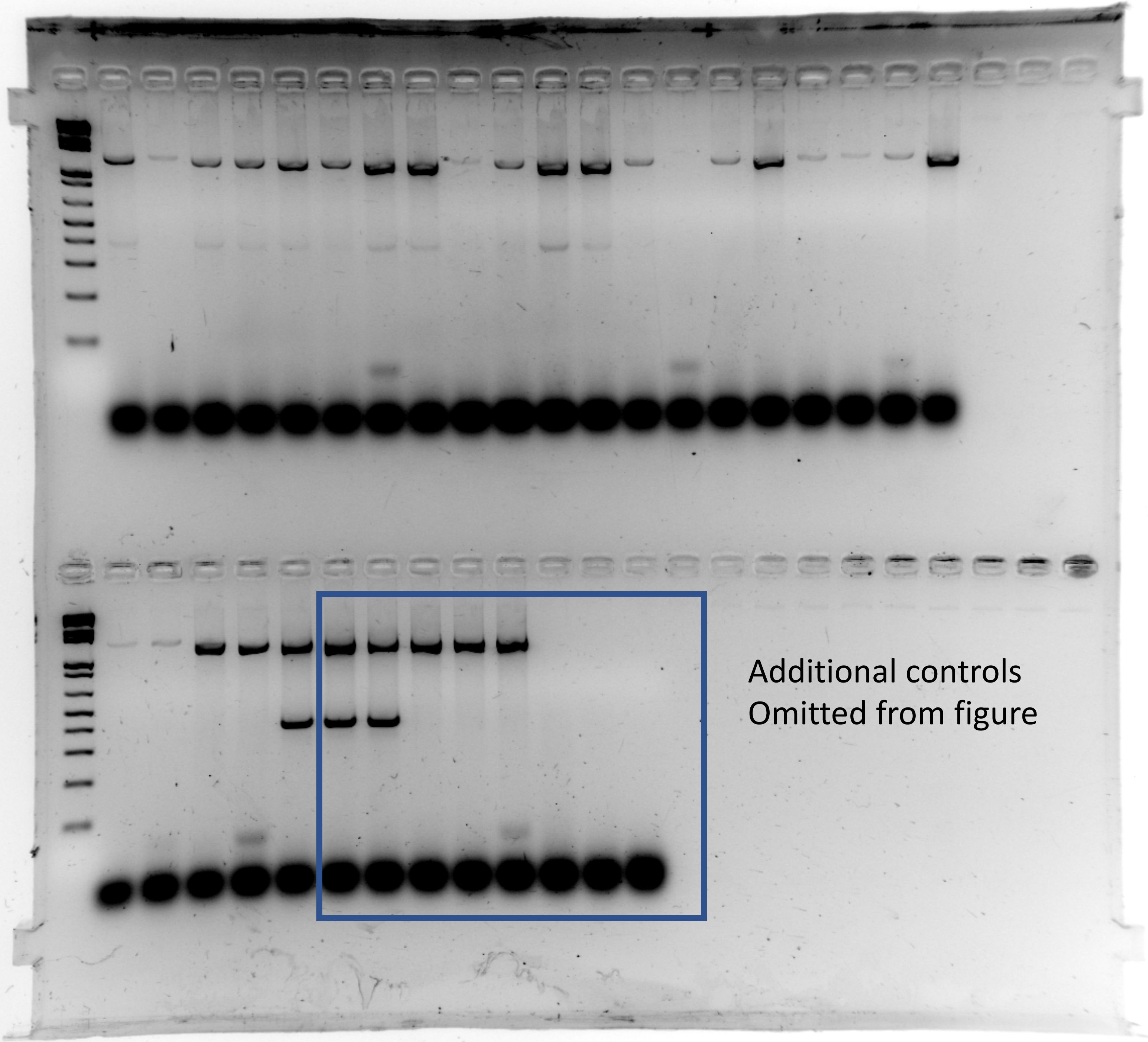

Uncut gel for Supplementary Fig 1

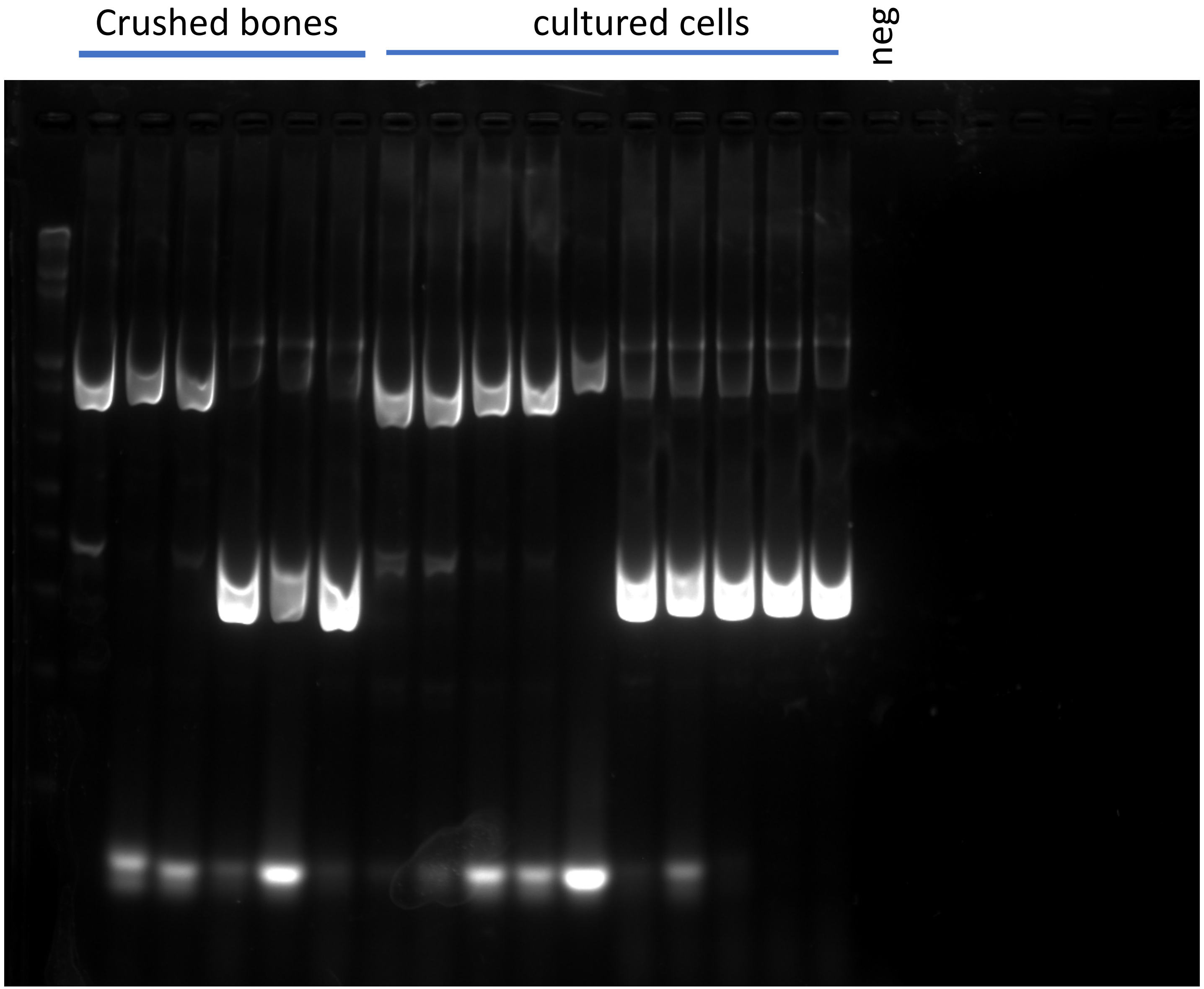

Supplement: Supplementary file 1 — Supplementary Information. [file 41598_2022_8914_MOESM1_ESM.pdf]
